# Cross-cultural comparison of the patient-centeredness of the hidden curriculum between a Saudi Arabian and 9 US medical schools

**DOI:** 10.3885/meo.2009.T0000144

**Published:** 2009-12-18

**Authors:** Rasha Al-Bawardy, Benjamin Blatt, Saad Al-Shohaib, Samuel J. Simmens

**Affiliations:** * Department of Medicine, School of Medicine and Health Sciences, The George Washington University, Washington, D.C., United States of America; † Department of Medicine, King Abdulaziz University, Jeddah, Saudi Arabia; ‡ Department of Epidemiology and Biostatistics, School of Public Health and Health Services, The George Washington University, Washington, D.C., United States of America

**Keywords:** medical education, hidden curriculum, cross-cultural comparison, patient-centered care

## Abstract

**Background:**

The implicit “hidden curriculum” strongly influences medical students’ perceptions of the importance of patient-centeredness. A new instrument, the Communication, Curriculum, and Culture Survey (C3), already used to assess this hard-to- access part of the curriculum in the US, has potential for use in cross-cultural comparisons.

**Objective:**

To use the C3 to perform a pilot cross-cultural comparison of the patient-centeredness of the hidden curriculum between a Saudi medical school and 9 U.S. medical schools.

**Design:**

Senior Saudi medical students completed the C3 and a second instrument, the Patient-Provider Orientation Scale (PPOS), which measured their attitudes toward patient-centered behavior.

**Participants:**

Senior Saudi medical students.

**Results:**

139/256 (54%) Saudis completed the C3; 122/256(48%) completed the PPOS. Means for 2 out of 3 of the C3's domains (0–100 scale) were lower for the Saudis than those for the Americans (95% confidence intervals in parentheses): 47 (45, 50) vs. 55 (53, 58); 54 (50, 58) vs. 68 (67, 70); they overlapped in the third: 60 (57, 63) vs. 62 (60, 63). The mean Saudi PPOS score was 4.0 (3.9, 4.1); for the American medical schools, 4.8 (4.8–4.8) (1–6, least to most patient-centered).

**Conclusions:**

In this preliminary study the data suggest that the patient-centeredness of the hidden curriculum differs in Saudi and US medical schools in 2 out of 3 domains. Cross-cultural use of instruments such as the C3 can highlight such important differences and help educators evaluate their currciulum from an international, as well as a local perspective. Use of instruments across borders is a growing trend and an indicator of the increasing globalization of medical education.

## Background

In recent years, the formal curriculum of US medical schools has stressed patient-centered care, encouraged by leading organizations in medical education.^1^ Supporting this trend, Western studies have shown that physicians trained in patient-centeredness tend to be more compassionate, humanistic and relate better with patients.^2^, ^3^ Medical schools introduced patient-centeredness into the formal curriculum of US medical schools in the form of ethics, communication, and humanities courses and in Hippocratic Oath and “white coat” ceremonies. Studies have shown, however, that such activities do not necessarily promote more patient-centered behaviors among medical students.^2^ This may be because the actual learning environment experienced by medical students, sometimes called the hidden curriculum, undermines their patient-centeredness.^4^ The hidden curriculum includes “stories, jokes, and personal anecdotes, [from] faculty or fellow students, [which] function as part of the oral culture of medical training.”5 (p. 865)

The hidden curriculum was at first studied ethnographically, uncovering discrepancies between ideal and actual behavior.^6^ Ethnographic studies, however, are time-intensive and difficult to perform. Recently, the Communication, Curriculum, and Culture Survey (C3) was developed and validated, providing easier access to the hidden curriculum by probing students’ experience. In 2006 Haidet et al. reported the results of surveying 9 U.S. medical schools using the C3. They “demonstrated unique and different learning environments both in terms of magnitude and patterns characteristics.”7 (p.405)

As far as we know, the C3 has never been used to characterize the hidden curriculum of medical schools outside the United States. In this pilot study we administered the C3 to 6^th^ year medical students (equivalent to senior U.S. medical students) at King Abdul Aziz University (KAAU) in Jeddah, one of five major Saudi medical schools. Our primary purpose is to report these preliminary results and compare them to those from the 9 U.S. medical schools.^7^ To supplement this cross-cultural comparison, we also examined the personal patient-centeredness of Saudi and American students using a validated survey, the Patient-Provider Orientation Scale (PPOS).^6^

## Methods

**Participants** – With KAAU Medical School and the GW IRB approval, we asked Saudi medical students in their 6^th^ (last) year of medical school to complete both the C3 and the Patient-Provider Orientation Scale (PPOS). One of the authors of this paper (SA), a KAAU faculty member, notes that Saudi students accept surveys as part of their medical training and commonly complete them.

**Instruments** – The survey was translated by this same KAAU faculty member (SA). He trained in nephrology in Canada and is fluent in both English and Arabic. The survey presented the respondents with each question in both English and Arabic. Admission to the KAAU School of Medicine requires candidates to be fluent in both written and spoken English, since the curriculum is taught in English. It was thus thought that respondents would be able to accurately grasp the full meaning of each question. The survey was handed out in class to 100% of the 6^th^ year students.

**C3** – The C3 survey measures three content areas of hidden curriculum patient-centeredness:

1. Role modeling – by faculty/residents
2. Student experiences – of instances of non-patient centered behaviors
3. Support for students’ patient-centered behavior

The instrument consists of 29 items scaled from 1–7 in content area 1, and from 1–5 in content areas 2 and 3.^1^ (Higher numbers indicate a more patient-centered score.) All C3 score statistics are presented based on transformation of raw scores to a possible 0 to 100 range.

Sample items from each content area of the C3:

*Role Modeling*: “Please indicate how often you observed senior residents communicate concern and interest in patients as unique persons” (always, almost always, more than half the time, less than half the time, rarely, never)

*Students’ Experiences*: “You hear an attending physician discussing a patient's case history with another attending or house officer. During the course of the conversation, the patient is referred to as a diagnosis (e.g., ‘I had a great pancreatitis case on my team the other day’). Rate how often you have experienced a similar situation” (very often, fairly often, occasionally, rarely, never)

*Support for Students’ Behaviors*: “In general, when I made an effort to legitimize patients’ concerns about their condition or care, my instructors’ ____________ me.” (completely encouraged, mostly encouraged, slightly encouraged, neither encouraged or discouraged, discouraged)

**Patient- Provider Orientation Scale** – The PPOS consists of 18 items rated by students using a six-point Likert-scale response. The PPOS has two main categories: “sharing,” and “caring.” The 9 “sharing” questions target beliefs in patient-physician power and control sharing; the 9 “caring” questions address warmth and support in the patient-physician relationship. The overall score is calculated as the mean of the individual scores (1= most “doctor-centered;” 6, most “patient-centered”).^6^

Sample items from the PPOS:

*Sharing:* The doctor is the one who should decide what gets talked about during a visit.

*Caring:* If doctors are truly good at diagnosis and treatment, the way they relate to patients is not that important.

**Data Analysis** – Descriptive statistics, including confidence intervals, were calculated for the Saudi student responses. For the American medical school participants, descriptive statistics for the PPOS are taken with permission from Haidet et al (2005)^1^ and C3 score descriptive statistics from Haidet et al.(2006).^7^ For the latter, the results were presented separately for each of nine medical schools after adjusting for age, gender, attitudes towards patient-centered care, and clinical experience.^7^ Haidet reports that these adjustments had minimal impact on the descriptive results (2007, email from author; unreferenced). Nonetheless, we could not perform a significance test comparing the Saudi and American C3 score results as the former is unadjusted and the latter adjusted. Instead, we emphasized confidence intervals as displayed in Table 1. For the PPOS findings, the Haidet results are unadjusted, allowing a 2-sample t-test to compare the American and Saudi means.

**Table 1.** Comparison of Communication, Curriculum, and Culture Survey (C3) Results, 9 U.S. Medical Schools vs. Saudi Medical School Results are reported on a 0–100 scale (100 best). Confidence intervals are in parentheses

	C3 Score: Role Modeling	C3 Score: Students’ Experiences	C3 Score: Support for Students’ Behaviors
U.S. Medical Schools	61.7 (60,63)	55.2 (53,58)	68.4 (67,70)
Saudi Medical School (KAAU)	59.9 (57,63)	47.3 (45,50)	54.3 (50,58)

## Results

Fifty-four percent of students (139/256) completed the C3 survey and forty-eight percent (122/256) completed the PPOS survey. Participants included both genders and all were Saudi citizens, sharing the same culture and religion.

Means for the C3 instrument's 3 content areas, on a 0–100 scale, were lower in 2 out of 3 domains for the KAAU medical school compared to the average of the 9 American medical schools: Student Experiences in Patient-Centered care–47 (CI = 45,50) vs. 55 (CI = 53,58); and Support for Students’ Own Patient-Centered Behaviors–54 (CI = 50,58) vs. 68 (CI = 67,70). The third domain, Role Modeling, received similar scores in both countries, 60 (CI = 57,63) vs. 62 (CI = 60,63). For Role modeling, however, the KAAU mean was lower than 7 of 9 American medical schools, while the other two measures the KAAU mean was lower than all 9 American medical schools. Coefficient alphas for Student Experiences in Patient-Centered Care, Support of Students Own Patient-Centered Behavior, and Role-Modeling were .60, .73, and .90, respectively. Coefficient alphas for these content areas for the 9 American medical schools were .69, .85 and .93 respectively.^1^

As noted in Table 2, the mean PPOS score for the Saudi cohort was 4.0 (CI = 3.91, 4.09), compared with 4.8 (CI = 4.76–4.84) in American medical schools (1–6 representing least to most patient-centered). Coefficient alphas were .56 (Total), .38 (Sharing) and .46 (Caring). In previous research using the PPOS, the co-efficient alphas ranged from .75 to .88.^8^

**Table 2.** Comparison of Patient-Provider Orientation Scale (PPOS) Results, 9 U.S. Medical Schools vs. Saudi Medical School Results are reported on a 1–6 scale (6 best). Confidence intervals are in parentheses

	PPOS Score
9 U.S. Medical Schools	4.8 (4.76–4.84)
Saudi Medical School (KAAU)	4.0 (3.91, 4.09)

Gender differences were tested for both the C3 and PPOS through t-tests. Although females tended to have higher means on 4 of the scales (Role Modeling, Student Experiences, PPOS Total, and PPOS Sharing), the only statistically significant difference was for PPOS sharing (p=.001), with female students indicating more sharing (mean = 4.12; sd=.53) compared to the male students (mean = 4.00; sd=.44). The questionnaires of twenty-eight students were missing gender designations, and therefore are excluded from these analyses.

## Discussion

The data in this preliminary study suggest that there may be cultural differences in the hidden curricula of the Saudi and American medical schools. The difference in Saudi and American students’ scores in the Student Experiences in Patient-Centered Care and Support for Students’ Own Patient-Centered Behaviors survey categories suggests that the hidden curriculum in the Saudi School is more physician- and less patient-centered than in the American medical schools in these 2 domains. Results were similar for American and Saudi students for the role modelling domain. Results did not differ across genders. If the Saudi-American difference in hidden curriculum were to be confirmed by a larger survey, it would not be surprising as differences exist in Saudi and American cultural norms. Mobeireek et al. comment in their study of Saudi physicians’ communication: “In traditional societies [like Saudi Arabia] where physicians are regarded as figures of authority and family ties are important, there is a considerable shift of access to information and decision-making from patients to their physicians and relatives in a manner that threatens patients’ autonomy.”9 (pg.284) Also reflecting the influences of traditional Saudi values are the PPOS scores of Saudi students, which show more physician-centeredness than their American counterparts. As discussed below, however, Saudi society is neither monolithic nor static, and evidence exists of movement toward more patient autonomy as exemplified by the increase in patient-initiated medical litigation.^10^

Because traditional Saudi values are overtly physician-centered, we hypothesized that the Saudi formal curriculum would also be physician-centered. As a result, we expected great similarity between the formal and the hidden curriculum in Saudi medical schools. Our expectations were supported by Elzubier's^11^ comments regarding the lack of teaching patient-centered values in the Saudi formal curriculum: “In Saudi Arabia, the acquisition of the skill of doctor-patient communication hardly exists in any undergraduate or post-graduate medical curriculum.” The experience of one of our researchers (SA) supports Elzubier's view. If these perceptions are accurate, what is professed and what is done in Saudi medical education may be similar. Without a mismatch between these two elements, there is no hidden curriculum in regards to patient-centeredness. The situation, however, may be more complex. Though almost all of the KAAU medical school faculty are Saudi, many trained in Western countries and may have acquired Western outlooks. Saudi students are thus exposed to influences that may be more patient-centered than the traditional Saudi curriculum. Further research is needed to gain a more nuanced picture of the Saudi curriculum, both hidden and overt, and Saudi students’ attitudes and responsiveness toward this complex set of influences.

The C3, in presenting an window into the patient-centeredness of the hidden curriculum, allows educators to assess it. One obvious standard by which to judge it is the compatibility with the needs of Saudi society. Since traditional Saudi social values tend toward paternalism, it is easy to assume a happy congruence between the physician-centered medical curriculum and society's needs. However, Saudi society, like many traditional societies exposed to globalization^12^, ^13^, ^14^, ^15^, is complex and changing. In the last 30 years it has been moving from a nomadic to an urbanized existence.^9^ Urbanization, with ready internet access to medical information, catalyzes patient desire for autonomy and patient-centeredness. We know of no data from which we can derive a comprehensive picture of the expectations of the Saudi population for patient-centered care. The closest we can come are studies of patient satisfaction with the Saudi health care system.^16^, ^17^ In one of these studies,^17^ physician's “attentive listening to patient complaints,” a marker for patient-centered care, was scored poorly by patients. Without studies that sample a broad range of the Saudi population (e.g., urban vs. rural, different ethnicities) on their expectations, however, it is not possible to draw valid conclusions. That being said, the Saudi patient movement toward autonomy parallels a similar movement which occurred in the United States 50 years ago and which has occurred or is occurring now in many other countries.^18^,^19^,^20^,^21^,^22^ Medical educators across the globe from very different traditions are presented with a very similar challenge: how to best train their students to work in partnership with patients who are moving towards greater autonomy and who may have very different expectations from those of the past. For medical educators throughout the world, a benefit of being confronted by this common challenge is the opportunity for international dialogue, collaboration and research on such topics as those presented in this paper.

This pilot effort has important limitations. First, because 54% of the student population chose to respond to the survey and because it was conducted in one Saudi medical school, the results may not be representative of all Saudi students. This report, however, was conceived as a preliminary description “from” to of a pilot study, with the intention of extending it to other Saudi schools in the future. Second, special procedures were not undertaken to verify that the meaning of the survey questions were maintained across cultures. Since we presented English-fluent students with surveys in English with a Saudi translation beneath each item, we assumed the meaning would be clear. It would have been better to have verified the stability of the meaning through consensus of bilingual evaluators. Third, the internal consistency of the PPOS was considerably lower in this study than in studies with American subjects. Possible explanations include lack of reliability of this instrument in this particular culture or the introduction of random error from such sources as students’ misunderstanding of survey items.

## Conclusions

The use of the C3 cross-culturally is an example of a growing trend toward the globalization of medical education evaluation. The preliminary results presented here suggest that instruments validated in one country may be used in other countries to generate important cross-cultural insights. The C3 may provide a means for both Saudi and American educators to gauge the patient-centeredness of their curricula, compare them internationally, and then decide, within their own cultural and educational contexts, whether alterations should be made. The C3 also may be useful for Saudi educators if the formal Saudi curriculum follows trends within Saudi society and becomes more patient-centered. If the hidden curriculum does not follow suit but remains physician-centered, serial administration of the C3 could alert Saudi educators to this discrepancy so they could take appropriate action to minimize it.
